# *Cryptosporidium andersoni as* a novel predominant *Cryptosporidium* species in outpatients with diarrhea in Jiangsu Province, China

**DOI:** 10.1186/s12879-014-0555-7

**Published:** 2014-10-25

**Authors:** Yanyan Jiang, Jinhua Ren, Zhongying Yuan, Aiqin Liu, Hong Zhao, Hua Liu, Lei Chu, Wei Pan, Jianping Cao, Yijin Lin, Yujuan Shen

**Affiliations:** National Institute of Parasitic Diseases, Chinese Center for Disease Control and Prevention; Key Laboratory of Parasite and Vector Biology, Ministry of Health, WHO Collaborating Center for Malaria, Schistosomiasis and Filariasis, Shanghai, 200025 China; Department of Infectious Diseases, Danyang Hospital of Jiangsu Province, Danyang, 212300 China; Department of Parasitology, Harbin Medical University, Harbin, 150081 China

**Keywords:** Cryptosporidium andersoni, Diarrhea, Stool specimens, Predominant species, Prevalence

## Abstract

**Background:**

*Cryptosporidium hominis* and *C. parvum* are usually considered to be the major pathogens responsible for human cryptosporidiosis. However, there have been few studies regarding the molecular epidemiology of *Cryptosporidium* in human infections in China. Here we investigated *Cryptosporidium* infection in patients with diarrhea, in Danyang Hospital of Jiangsu Province, China, at the genotype level.

**Methods:**

A total of 232 stool specimens were collected from outpatients with diarrhea in Danyang Hospital of Jiangsu Province, China, from February 2012 to January 2013. Each specimen was stained from direct fecal smears and examined for *Cryptosporidium* using modified acid fast staining and microscopy. Moreover, genomic DNA of each fecal sample was screened for the presence of *Cryptosporidium* with nested PCR, which was genotyped by analyzing the DNA sequences of small subunit rRNA (SSU rRNA).

**Results:**

The average infection rate of *Cryptosporidium* was 1.3% (3/232) by microscopy and subjected to PCR amplification of the SSU rRNA gene of *Cryptosporidium*, with 9.91% (23/232) being positive for *Cryptosporidium* with a significant peak in autumn. Based on the SSU rRNA gene, two *Cryptosporidium* spp. were identified, including *C. andersoni* (n =21) and *C. hominis* (n =2). Two types of *C. andersoni,* designated as A_370_^+^ and A_370_^-^ , were found in the SSU rRNA gene in our present study, which was 100% homologous to *C. andersoni* infections derived from dairy calves and goats, respectively. The clinical questionnaires showed no significant difference in age, gender and frequency of diarrhea, but duration of diarrhea was shorter for *C. andersoni* than that of *C. hominis* (mean, 2 vs. 4 days; *p* <0.01).

**Conclusions:**

*C. andersoni* is the dominant species in Danyang City of Jiangsu Province. The fact that SSU rRNA sequences of *C. andersoni* obtained from human stools exhibited 100% homologous to those derived from dairy calves and goats supported that *C. andersoni* infection might be attributable to animal origin. The difference in the duration of diarrhea of *C. andersoni* and *C. hominis* indicated that different *Cryptosporidium* species might cause different clinical manifestations.

## Background

*Cryptosporidium* spp. causes significant diarrheal disease in humans and animals worldwide [1]. Increasing numbers of molecular epidemiological studies on cryptosporidiosis have provided greater understanding of the diversity of the species infecting humans. To date, 30 species and more than 70 subtypes of *Cryptosporidium* have been identified in diverse vertebrate hosts [2]-[4]. Among these, *C. hominis* and *C. parvum* have been shown to be the two major human pathogens by numerous studies, responsible for approximate 90% of human cryptosporidiosis [5]-[8]. Moreover, there appear to be geographical differences in the distribution of *Cryptosporidium* spp. in humans from an epidemiological viewpoint. For example, *C. parvum* is the dominant species infecting humans in the Middle-East, while *C. hominis* is considered to be responsible for the majority of human infections in developing countries, and *C. parvum* and *C. hominis* are both observed in almost equal proportions in European countries [1],[9].

In China, since the first report of two human cases of cryptosporidiosis in 1987 [10], *Cryptosporidium* has been attracting increasing attention. Epidemiological data on human cryptosporidiosis have confirmed its common occurrence in at least 17 Chinese provinces or cities with a detection rate ranging from 1.4% to 10.4% based on morphological identification [11]. Identifying the parasite species infecting humans by molecular methods is important in determining the epidemiology of the disease and its likely transmission routes [12]. However there have been few studies conducted on the molecular epidemiology of *Cryptosporidium* in humans, with the exception of Shanghai [13], Henan Province [14], Hunan Province [15] and Tianjin [16]. Currently, there are only morphological data on human cryptosporidiosis in Jiangsu Province [17],[18]. The aim of the present study was to investigate *Cryptosporidium* infection in patients with diarrhea using molecular tools and sequencing, in Danyang Hospital of Jiangsu Province. We then further sought to investigate the possible relationship between epidemiological data and clinical manifestations of the predominant *Cryptosporidium* species detected.

## Methods

### Ethics statement

Ethical approval for the collection and examination of human feces samples was obtained from the Ethics Committee of the Danyang Hospital of Jiangsu Province and the National Institute of Parasitic Diseases, Chinese Center for Disease Control and Prevention, China (reference no. 2012-12). The objectives, procedures and potential risks were verbally explained to all participants. Written informed consent was given to, and signed by all the study participants. Parents/guardians provided consent on behalf of all infant participants.

### Fecal specimen collection and examination

Human stool specimens (n =232) were collected from Danyang Hospital of Jiangsu Province, China. All of the diarrhea patients investigated (1 month - 77 years old, medium 34.7 years old) in this study were recruited from outpatients departments during a 1-year period from February 2012 to January 2013. Patient details, including their age, gender, occurrence, duration and frequency of diarrhea, and consistency of stools, were recorded. The duration of an episode diarrhea was defined to be from the onset to attendance at the hospital. The frequency of diarrhea episodes was defined as the number of occurrences of diarrhea per day. Specimens were collected from patients with fecal excretion heavier than 200 mg and with no less than three events of diarrhea per day. Each specimen was stained from direct fecal smears and examined for *Cryptosporidium* using modified acid fast staining and microscopy [19],[20].

### DNA extraction

Sufficient samples (200 mg of each fecal sample) were collected for DNA extraction and purification using a commercially available kit, QIAamp DNA Mini Stool Kit (Qiagen, Valencia, CA). The extracted DNA samples were stored at –30°C for PCR.

### Genotyping of *Cryptosporidium*

All DNA preparations were screened for the presence of *Cryptosporidium* DNA by nested PCR amplification of a fragment (approximately 830 bp) of the SSU rRNA gene, and were identified to the species/genotype level as previously described [21],[22].

Each DNA preparation was performed three times by using 1 μl DNA per PCR. DNA from *C. baileyi* obtained from a chicken [23], provided by Prof. Longxian Zhang of Henan Agricultural University, was used as a positive control, and deionized water was used as a negative control. All nested PCR products were visualized by electrophoresis in 2% agarose gel stained with ethidium bromide before sequencing.

### DNA sequence analysis

Positive secondary PCR products were subjected to two-directional sequencing with secondary primers by the Shanghai Sunny Biotechnology Co, Ltd. (Shanghai, China). The accuracy of the sequencing data was confirmed by sequencing in both directions. Amplified sequences were blasted against sequences in the NCBI database and then deposited in GenBank (accession numbers KF826294 to KF826316) and identified by direct comparisons using the MEGA5 software of the acquired nucleotide sequences with each other and with reference sequences downloaded from GenBank.

### Statistical analysis

Significance testing was performed using χ^2^ or Fishers exact test for categorical variables. Differences were considered significant at *p* values of <0.05. All statistical analyses were performed using JMP software (SAS Institute, USA).

## Results

### Prevalence of cryptosporidiosis in patients with diarrhea in Jiangsu Province

Human stool specimens (n =232) were screened microscopically for the presence of *Cryptosporidium* oocysts. However, the average detection rate of *Cryptosporidium* was only 1.3% (3/232) by microscopy with average dimension of the *Cryptosporidium* oocysts of 7 μm × 5 μm. These specimens were also screened for the presence of *Cryptosporidium* by PCR (9.9%) collected from February 2012 to January 2013 in Danyang Hospital of Jiangsu Province (Table 1). The prevalence of *Cryptosporidium* in different seasons was also investigated using these specimens. The mean prevalence over the four seasons ranged from 2.2% to 29.1% and showed significant variation between seasons (χ^2^ = 9.753, *p* <0.05) (Table 1), with a peak in autumn. The prevalence of *Cryptosporidium* in all age categories (with the exception of children group) ranged from 7.7% to 12.5%, with no significant differences among different age groups (χ^2^ = 1.666, *p* >0.05) (Table 2).

**Table 1:** **The seasonal distribution of cryptosporidiosis in diarrhea outpatients in the Danyang hospital of Jiangsu province from February 2012 to January 2013**

Season	No. specimens	No. positive(%)	Species	
			*C. andersoni*	*C. hominis*
Spring	32	1(3.1)	0	1
Summer	99	5(5.1)	5	0
Autumn	55	16(29.1)^b^	15	1
Winter	46	1(2.2)	1	0
Total	232	23(9.9)^a^	21	2

NOTE. All specimens were collected from February 2012 to January 2013 (divided into four seasons) and *Cryptosporidium* species were detected by the SSU rRNA gene analysis*.*

^a^The prevalence of *Cryptosporidium* infection.

^b^Data from autumn show a significant difference compared with those of other seasons (χ^2^ = 9.753, *p* <0.05).

**Table 2:** **Feature distribution of** ***Cryptosporidium*** **spp. and subtype in Danyang hospital of Jiangsu province from February 2012 to January 2013**

Feature	No. samples	No. positive(%)	*C. andersoni*(%)	*C. hominis*
**Cases**	232	23 (9.9)	21 (91.3)	2 (8.7)
**Age category(yr)** ^**a**^				
Infants (<5 years), n (% )	66	7 (10.7)	7 (33.3)	0
Children (5-12 years), n (%)	6	0 (0. 0)	0	0
Youths (13-19 years), n (%)	10	1 (10.0)	1 (4.8)	0
Adults (20-49), n (%)	78	6 (7.7)	5 (23.8)	1
Elderly (50 years up), n (%)	72	9 (12.5)	8 (38.1)	1
**Gender**				
Female	94	5 (5.3)	4 (19.1)	1 (50.0%)
Male	138	18 (13.0)	17 (81.0)	1 (50.0%)
**Infection episode**				
Duration of diarrhea, days	4	3	2^b^	4
Frequency of diarrhea, times	6	5	5	3.5

NOTE. All specimens were detected by the SSU rRNA gene analysis of *Cryptosporidium* species. ^a^There was no significant difference in the prevalence of *Cryptosporidium* among all age categories (χ^2^ = 1.666, *p* >0.05). ^b^Statistically significant at *p* <0.01 with *C. andersoni* infection (n =21) *vs. C. hominis* infection (n =2).

### Molecular analysis of *Cryptosporidium*species

Nested PCR screening for *Cryptosporidium* identified 23 positive specimens. DNA sequencing of the SSU rRNA target fragments revealed the presence of two *Cryptosporidium* species: *C. hominis* (n =2) and *C. andersoni* (n =21) (Table 1). All three microscopy-positive specimens were further confirmed to be *C. andersoni* by PCR and sequencing of the SSU rRNA gene. In addition, two distinct *C. andersoni* sequences were classified at the SSU rRNA locus. Of the 21 SSU rRNA *C. andersoni* sequences obtained (771 bp in length), 12 sequences were assigned as A_370_^−^ type (KF826294, KF826295, KF826296, KF826298, KF826299, KF826303, KF826304, KF826305, KF826307, KF826310, KF826311, KF826313) and nine sequences as A_370_^+^ type (KF826297, KF826300, KF826301, KF826302, KF826306, KF826308, KF826309, KF826312, KF826314), based on the absence or presence, respectively, of an A at position 370 of the nucleotide sequence. A_370_^−^ type exhibited 100% homology with the *C. andersoni* isolated from dairy calves (JX515549), while A_370_^+^ type exhibited 100% homology with the *C. andersoni* isolated from a goat (EU926593).

### Characteristics associated with *C. andersoni*infection

According to *Cryptosporidium* infection among these age groups, *C. hominis* infection was found in two specimens from the adult group and elderly group, respectively, with an overall detection rate of 8.7% (Table 2). In contrast, *C. andersoni* infection was more commonly found in diarrheal patients, with the exception of children (Table 2). There were no significant differences in *C. andersoni* detection rates between the different age groups (χ^2^ = 1.82, *p* >0.05). However, according to our data, the detection rate of *C. andersoni* infection was 33.3% in infants and 38.1% in elderly patients. Further analysis of the results showed no significant difference in the overall number of *Cryptosporidium*-positive specimens between the genders (χ^2^ = 3.74, *p* >0.05); however the gender distribution of *C. andersoni* infection was four (19.05%) female and 17 (81.0%) male patients, compared with one female (KF826315) and one male patient (KF826316) for *C. hominis* (Table 2).

Analysis of associated information reported revealed that the outpatients with *C. andersoni* attended the hospital after a shorter duration of diarrhea compared to those with *C. hominis* infection (mean, 2 days and 4 days, respectively; *p* <0.01). Although there was no significant difference in the frequency of diarrhea episodes between these two species, we noticed a tendency for *C. andersoni* to result in more serious diarrhea, with a mean frequency of five times per day (Table 2).

## Discussion

Knowledge of *Cryptosporidium* species is essential to the management of cryptosporidiosis and reduction in the risks of continued disease prevalence. In this study, the prevalence of *Cryptosporidium* spp. infection in our study was 1.3% according to microcopy versus 9.9% by PCR, and all three microscopy-positive specimens were confirmed as being *C. andersoni* by both PCR and sequencing of SSU rRNA gene. The result might be related to the fact that *C. andersoni* are larger than those of *C. hominis.* Meanwhile, no difference was observed morphologically between the dimensions of *C. andersoni* oocysts from humans in this study and those from animals reported previously [24]. Our molecular data was more sensitive and accurate than morphologic data, which was within the reported the range of 1.4%-10.4% of all patients with diarrhea for infections attributed to *Cryptosporidium* in China [10],[15]. However, our data for different age groups showed a detection rate of 12.5% in elderly patients, which was higher than that reported for elderly patients in Changchun (8.6%) [25]. This difference may be attributed to different detection methods in the same age group because PCR and microscopy detection were used in this study, while serological detection was used in the Changchun study. Our study indicates that molecular methods of detection have the advantage of increased sensitivity compared with conventional and immunological assays for detecting oocysts in feces. We also found an infection rate of 10.6% in infants in our study, which was higher than that of any pediatric hospital (0.4%-2.8%) in Shanghai [13]. These differences in prevalence may in part be related to differences in experimental design, specimen sources, and whether or not the patients were staying in hospital. In addition, our data also confirmed that seasonal variation can affect the prevalence of *Cryptosporidium*, with a peak in autumn [26],[27].

Although *C. hominis* and *C. parvum* account for more than 90% of human cases of cryptosporidiosis [2], other species that have also been associated with human disease globally include *C. meleagridis*, *C. cuniculus*, *C. ubiquitum*, *C. felis*, *C. canis* and *C. andersoni* as well as *C. muris*[5],[28]-[32]*.* There is considerable regional diversity in the distribution of *Cryptosporidium* species in China; *C. hominis* has been reported to be the predominant cause of infection in Eastern China [13],[16],[33], while *C. parvum* is the predominant species in Hunan Province [15]. However, the results of our study clearly showed that *C. andersoni* has become a novel predominant species (21 *C. andersoni* infections out of 23 cases) among outpatients with diarrhea in the geographical area investigated.

As a species with narrow host specificity [34], *C. andersoni* was usually detected primarily in domestic cattle [24]. Only a few cases have been published regarding human *C. andersoni* infections in France [35]*,* Malawi [36], Iran [37], England [5] and Australia [38]. However, a recent study by our group [39] reported 34 *C. andersoni* in 252 human patients with diarrhea in Shanghai. And in this study, we also found 21 cases of *C. andersoni* infection. It is unclear why so many cases of human cryptosporidiosis are caused by *C. andersoni* in the investigated areas. The true source of *C. andersoni* infection needs to be elucidated by subtyping *C. andersoni* isolates from humans and animals in the future, as well as analyses of its transmission dynamics. Furthermore, these sequences of the 21 cases of *C. andersoni* were divided into two types, which 12 sequences belonging to A_370_^-^ type were identical to an isolate from a dairy calf (JX515549), while the others of 9 sequences belonging to A_370_^+^ type were identical to an isolate from a goat (EU926593). This suggests that *C. andersoni* has become the new major *Cryptosporidium* species to infect humans, and its zoonotic transmission may be from animals to humans either directly or indirectly. We have already assessed further genetic sources of human *C. andersoni* infections using multilocus sequence typing (MLST), which has also been used for exploring genetic sources of *C. andersoni* infection in zoonotic samples [34],[40] and confirmed at least two sites from cattle to be consistent with our current data (data not shown).

*C. andersoni* is also one of the main *Cryptosporidium* species found in contaminated water such as the Potomac River in the United States [41], the Huangpu River in Shanghai [42], and in the urban wastewater plants in Harbin [43]. It is reasonable to suggest that *C. andersoni* infected humans could shed *C. andersoni* oocysts into urban wastewater, thus further contaminating water supplies and resulting in anthroponotic transmission of *C. andersoni*. It is therefore essential to conduct further investigation into the transmission dynamics of *Cryptosporidium* between dairy calves, humans and water resources in different geographic locations.

## Conclusion

In conclusion, the present findings, including distribution and genetic characterization of *Cryptosporidium* at the molecular level in patients with diarrhea, may be representative of the other molecular characteristics of human cryptosporidiosis. Whether or not the *C. andersoni* identified in patients with diarrhea in this study represented a natural infection needs to be confirmed with more systematic characterization of cryptosporidiosis and experimental infection studies.

## Abbreviations

*C. hominis*: *Cryptosporidium hominis*
*C. parvum*: *Cryptosporidium parvum*
*C. andersoni*: *Cryptosporidium andersoni*
SSU rRNA: Small subunit rRNA
